# Short-term efficacy and safety of percutaneous ultrasound-guided pseudomonas aeruginosa-mannose sensitive hemagglutinin injection in postoperative chyle fistula: a cohort study

**DOI:** 10.3389/fsurg.2026.1829380

**Published:** 2026-05-18

**Authors:** Yuhan Chen, Shouyi Yan, Tenghong Liu, Wenxin Zhao

**Affiliations:** 1 Department of Thyroid Surgery, Fujian Medical University Union Hospital, Fuzhou, Fujian, China; 2 Fujian Medical University, Fuzhou, Fujian, China; 3 Department of General Surgery, Fujian Medical University Union Hospital, Fuzhou, Fujian, China; 4 Clinical Research Center for Precision Management of Thyroid Cancer, Fujian Medical University Union Hospital, Fuzhou, Fujian, China

**Keywords:** chyle fistula, lateral neck dissection, papillary thyroid carcinoma, pseudomonas aeruginosa-mannose-sensitive hemagglutinin, ultrasound

## Abstract

**Objective:**

Postoperative chyle fistula is a rare but serious complication following total thyroidectomy with neck dissection, with an incidence of 3%–8% in high-risk patients. Given the variable effectiveness of conservative therapy, this study aimed to evaluate the short-term efficacy and safety of percutaneous ultrasound-guided pseudomonas aeruginosa–mannose-sensitive hemagglutinin (PA-MSHA) injection in postoperative patients.

**Methods:**

This retrospective single-center cohort study included 78 patients with papillary thyroid carcinoma who developed postoperative chyle fistula after total thyroidectomy with lateral neck dissection. Patients received either PA-MSHA injection (Group A) or standard conservative therapy including somatostatin and adhesion-agent injection (Group B). Inverse probability of treatment weighting (IPTW) was applied to balance baseline characteristics. Primary endpoints included postoperative length of stay (LOS), number of drug injections, readmission rates, daily drainage volume, and inflammatory responses. Secondary endpoints included side of leakage, unilateral drainage volume and demographic data.

**Results:**

All patients recovered without requiring surgical re-intervention. After IPTW adjustment, PA-MSHA treatment was significantly associated with a shorter LOS compared with conservative therapy (7.57 ± 1.49 vs. 8.86 ± 0.38). Daily clinical observations showed a visible reduction in chyle output within 24–48 h after PA-MSHA injection, the POD3/POD2 drainage reduction rate was 37% vs. 44%, not significantly different between groups after weighting, indicating a crude difference confounded by baseline drainage imbalance. Fever and neck pain were more frequent in PA-MSHA group, consistent with its inflammatory mechanism, and no severe complications occurred.

**Conclusion:**

Percutaneous ultrasound-guided PA-MSHA injection may serve as a practical adjunct to conservative therapy for postoperative chyle fistula, with a significant reduction in hospitalization duration after IPTW adjustment. Further prospective studies are needed to validate its efficacy and define its optimal clinical application.

## Introduction

Chyle fistula is a rare but potentially life-threatening complication following lateral neck dissection. The incidence of chyle fistula ranges from 0.5% to 9.7%, depending on the extent of surgery and variations in surgical protocols (1). This condition often leads to prolonged hospitalization and, if not managed properly, can result in serious consequences such as malnutrition, fluid and electrolyte imbalances, immune dysfunction, and skin necrosis (2–4). Surgical challenges associated with chyle fistula, such as intraoperative lymphatic damages, are particularly significant in high-risk patients, especially those with severe lymph node metastasis or obesity.

Initially, conservative treatments for chyle fistula including diet modification, continuous negative-pressure drainage, pressurized bandaging therapy, and fluid electrolyte compensation, are recommended. If conservative management fails or the chyle drainage exceeds 1,000 mL/day (classified as high-output), surgical intervention may be considered (5, 6). To prevent serious complications, Chinese guidelines recommend fine-needle aspiration cytology (FNAC) to assess lymph node metastases prior to surgery, along with lymphatic ligation during the procedure (7). However, the overall effectiveness of postoperative conservative treatments remains limited, with reported cure rates of approximately 66% in low-flow cases (<300 mL/day) and no definitive cure rate in high-flow cases (8). Despite numerous trials aimed at optimizing both preoperative evaluation and intraoperative prevention, standardized management protocols for chyle fistula have yet to be established.

**Figure 1:**
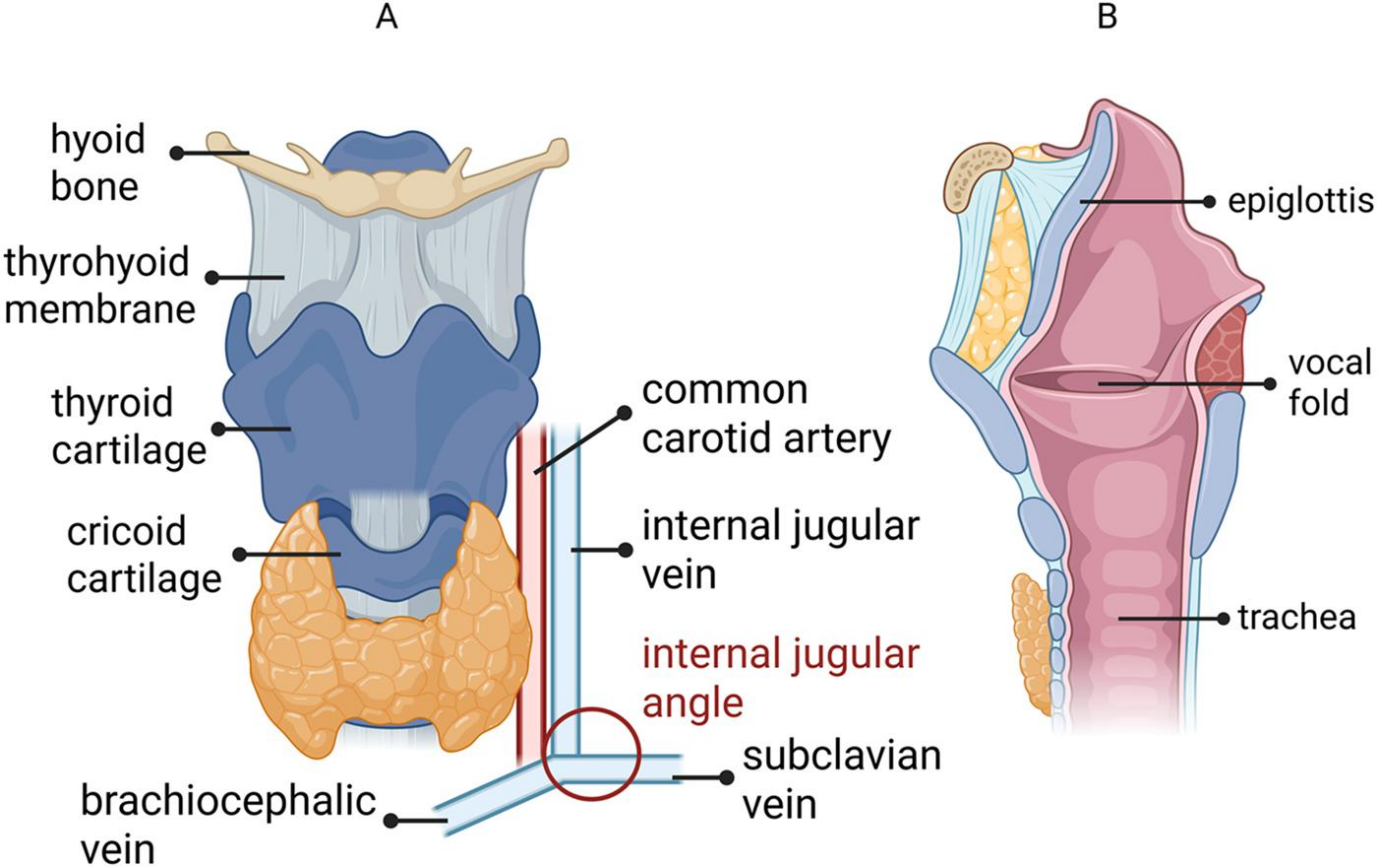
The anterior **(A)** and sagittal **(B)** anatomical views of the human larynx. The internal jugular angle, where the thoracic duct terminates, is the confluence of the internal jugular vein and the subclavian vein, playing a critical role in venous circulation. This figure was partially created using BioRender.com.

The thoracic duct, which carries lymph from the intestinal and lumbar lymphatics (9), ascends along the trachea and carotid artery, arching from the level of C7 to as high as C4, near the superior cornu of the thyroid cartilage (10). The internal jugular angle (Figure 1), where the thoracic duct terminates, is surrounded by a thin vessel wall and receives multiple venous circulations (11). Lymphatic leakage disrupts the vessel wall and infiltrates surrounding tissues, making the condition difficult to manage without external intervention. In addition to conservative approaches, PA-MSHA, a product derived from a genetically engineered strain of pseudomonas aeruginosa with multiple fimbriae on its surface (12, 13), has emerged as a promising treatment for chyle fistula following neck dissection (14). Individual case reports suggest that PA-MSHA injection following neck dissection results in a significant reduction in postoperative drainage volume within 12 h, outperforming continuous octreotide therapy. In cases where postoperative drainage remains between 300 and 400 mL/day, PA-MSHA injection has shown the potential to substantially reduce the drainage volume, providing an alternative to conventional treatments like octreotide therapy (13). However, limited large-scale studies have examined the use of PA-MSHA for chyle fistula, particularly after thyroid surgery.

The objective of this study was to evaluate the short-term efficacy and safety of percutaneous ultrasound-guided PA-MSHA injection in patients with chyle fistula. This study focused on the applicability and potential benefits of this approach in reducing postoperative complications following PTC surgery, improving patients' quality of life, and contributing to public consensus and satisfaction.

## Materials and methods

### Study population

From December 2018 to December 2020, a total of 78 patients diagnosed with papillary thyroid carcinoma (PTC) were enrolled in the study, where they were treated with total thyroidectomy plus lateral neck dissection (LND). The additional inclusion criteria were as follows: (1) No history of neck surgery; (2) Ipsilateral lymph node metastasis; (3) Drainage volume in the lateral neck region exceeding 100 mL on POD2; (4) Laboratory confirmation of a triglyceride concentration in the drainage fluid greater than 100 mg/dL.

**Figure 2:**
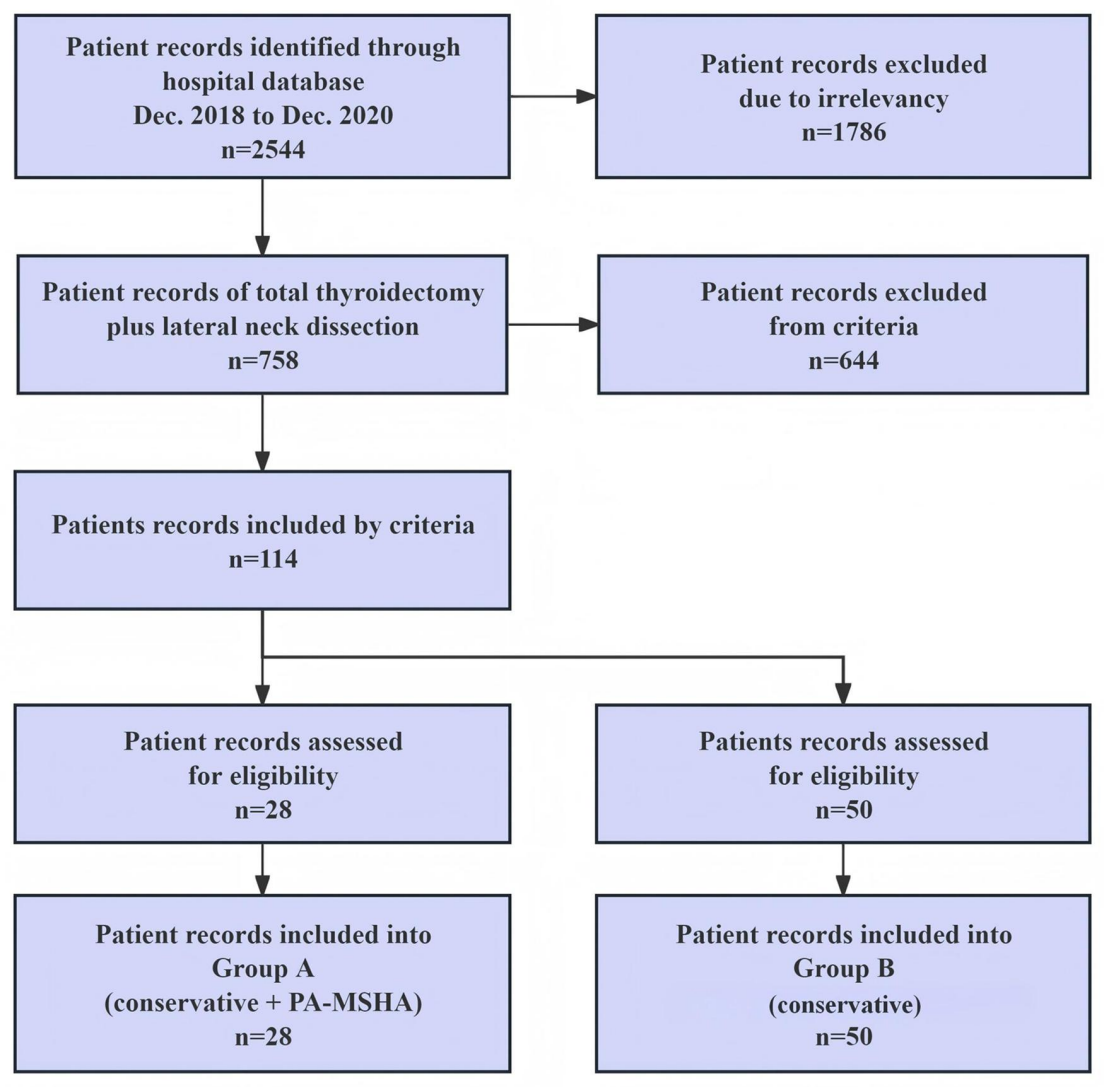
Study flow chart.

Eventually, all 78 patients were divided into two groups: Group A (received conservative therapy plus PA-MSHA injection) and Group B (received conservative therapy including external intervention, adhesion agent and somatostatin injection). The study flow chart is presented in Figure 2. This study was approved by the Ethics Committee of Fujian Medical University Union Hospital (grant number: 2019KY122). Informed consent was obtained from all participating patients. Patients in Group A were those who voluntarily agreed to receive PA-MSHA after informed consent, whereas patients who declined PA-MSHA received standard conservative therapy (Group B).

## Methods

**Figure 3:**
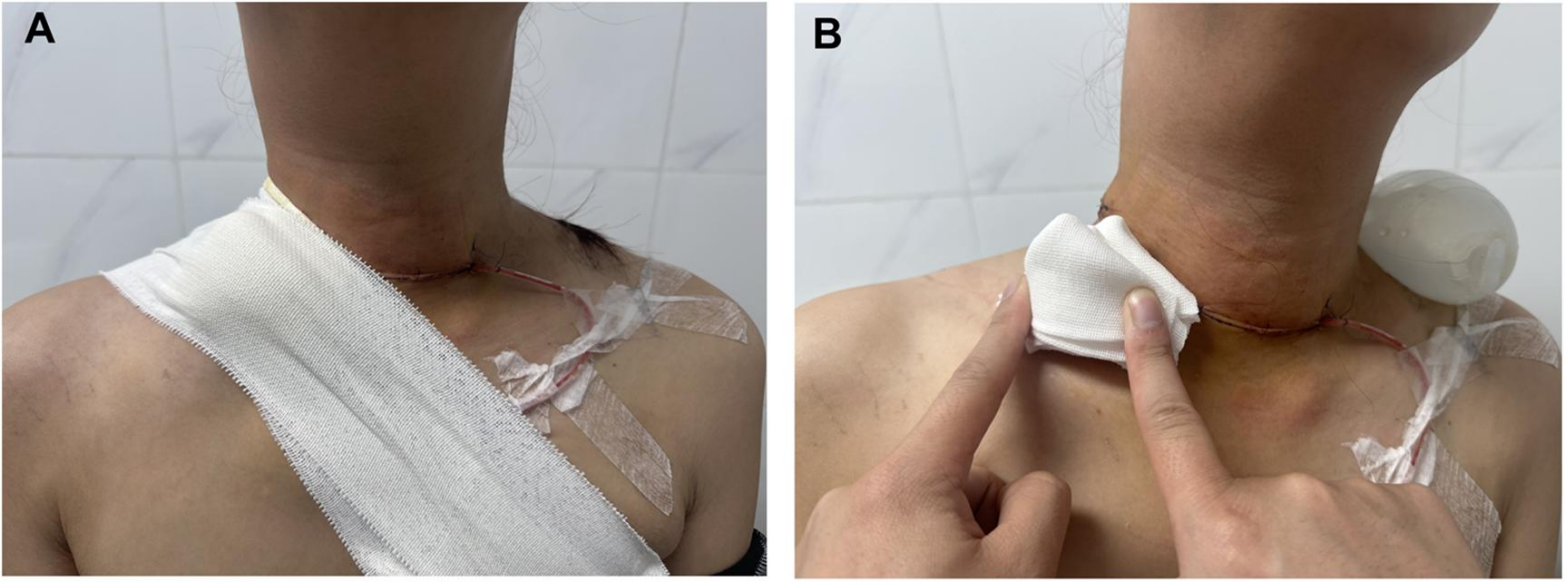
Postoperative PTC patients received conservative treatment including intravenous somatostatin and external intervention **(A)**. An overall view of the patient's neck wound and the basic drainage device. Pressurized bandaging was used in this study, folded into a triangle with the long edge aligned with the clavicle **(B)**.

All patients initially received conservative treatment, including diet modification, continuous negative-pressure drainage, pressurized bandaging therapy, and fluid and electrolyte compensation (Figure 3). The volume of chylous fluid drainage was measured every 24 h. Intravenous somatostatin therapy (6 mg every 12 h, twice daily, Longjing Pharmaceutical Co. Ltd, Kunming, China) was only administered to patients in Group B.

**Figure 4:**
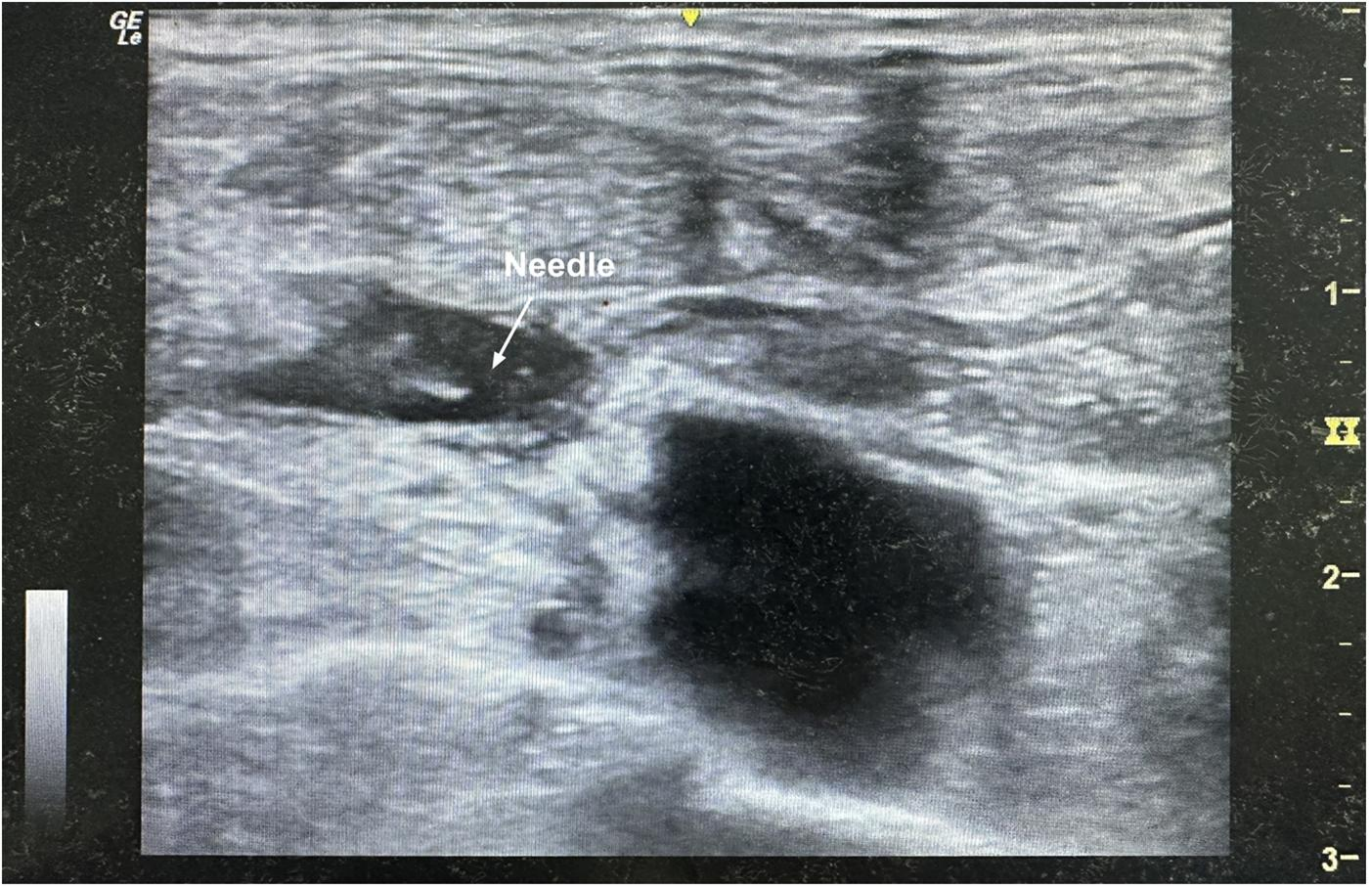
Real-time ultrasound image confirmed a percutaneous needle placement of PA-MSHA injection at the effusion site of postoperative PTC patients.

In Group A, percutaneous ultrasound-guided PA-MSHA injection (PA-MSHA, Beijing Wanter Bio-pharmaceutical Company, Beijing, China) was administered on the second day after surgery (POD2), based on conservative treatments (Figure 4). The injection procedure was as follows: First, the distal end of the tube was clamped for at least 15 min before PA-MSHA injection, primarily to clear the position of the lymphatic fistula. Next, the local effusion was suctioned out, and an undiluted 4 mL dose of PA-MSHA preparation (total bacterial count: 1.0 × 10^8^ cfu/mL) was injected into the space around the lymphatic fistula through the skin, guided by ultrasound, ensuring the preparation reached the site of the fistula. The distal end of the tube was then clamped for at least 30 min, followed by pressurized bandaging therapy applied to the supraclavicular skin. Noninvasive treatment was continued for the patients following the PA-MSHA injection.

In Group B, an adhesion agent (50% glucose or 10% meglumine diatrizoate) was injected into the space around the lymphatic fistula through the skin, guided by ultrasound. Somatostatin was considered necessary for patients with postoperative chyle fistula, which was also applied in our study. The two groups remained same baseline procedures and methods. Drainage volume on the third day after surgery (POD3) was measured for both groups to examine the short-term efficacy of PA-MSHA and conservative treatment. As drainage volume varied between individuals, a normalized proportion, referred to as POD3/POD2, was introduced to evaluate statistical differences between the two groups on a fair basis. Patients were considered recovered when the daily drainage volume showed a decreasing trend and remained below 30 mL/day for two consecutive days. Additionally, a “fatty meal” test was applied for each participant before drain removal. Repeat injections were considered if the drainage volume did not decrease by at least half of the second day's discharge within two days of the first injection. Surgical intervention was performed if drainage volume increased or persisted after the “fatty meal” test.

Ultrasound (US) assessment of the neck was conducted at 1 week and 1 month after drain removal. If necessary, a small amount of subcutaneous fluid was aspirated using a US-guided percutaneous puncture.

Primary endpoints included postoperative length of stay (LOS), the number of drug injections, the readmission rate, postoperative day 2 (POD2) and POD3 drainage volumes, normalized proportion of POD3 to POD2 drainage volumes (POD3/POD2 rate), and inflammatory responses such as fever and pain associated with PA-MSHA injection therapy. Secondary endpoints included total drainage volume (POD2 and POD3), side of leakage and unilateral drainage volume (POD2 and POD3), and demographic data.

To reduce baseline imbalance between two groups, a propensity score (PS) model was constructed. Propensity scores were estimated using a logistic regression model with treatment group (PA-MSHA vs. conservative) as the dependent variable and the following baseline covariates as predictors: age, sex, and POD2 total drainage volume. These variables were selected *a priori* based on their clinical relevance and availability before treatment initiation. Left/right drainage volumes were excluded to avoid collinearity with POD2 total drainage volume.

Inverse probability of treatment weighting (IPTW) was applied to create a weighted pseudo-population in which the distribution of baseline covariates was balanced between treatment groups. Stabilized weights were calculated as: 1/PS for Group A and 1/(1-PS) for Group B. The weighted sample approximates a randomized population in which treatment assignment is independent of measured baseline characteristics.

Covariate balance before and after weighting was evaluated using standardized mean differences (SMDs). An SMD close to zero indicates improved balance between groups. Weighted means and proportions were obtained using survey-weighted estimators. No outcome variables (fever, neck pain, number of drug injections) were included in the PS model because they occurred after treatment and were considered post-treatment adverse events.

After IPTW adjustment, treatment effects were estimated using weighted regression models. Continuous outcomes (LOS, total drainage volume, POD3/POD2 rate) were analyzed using weighted linear regression. Binary outcomes (fever, neck pain, number of drug injections) were analyzed using weighted logistic regression.

### Statistics analysis

Statistical analysis was performed using R (version 4.5.3), SPSS 27.0 (SPSS Inc., Chicago, IL, USA) and GraphPad Prism 10 (GraphPad Software, LLC). All values are expressed as the mean ± standard deviation. The *T*-test, Mann–Whitney *U*-test, ANOVA, and Chi-square tests were used to determine statistical significance. *P*-value of less than 0.05 was considered statistically significant. Figures were created using GraphPad Prism 10 (GraphPad Software, LLC) and BioRender.com.

## Result

### Patient characteristics and results

From a total of 758 patients who underwent total thyroidectomy plus lateral neck dissection (LND), 114 patients met the inclusion criteria. Eventually, 78 participants who provided informed consent were divided into Group A and Group B. Demographic characteristics and clinical procedures were summarized in Table 1. Among them, 28 patients in Group A received an additional percutaneous PA-MSHA injection, while the remaining 50 patients in Group B received a conservative therapy. Both interventions were based on noninvasive and conservative treatment principles.

**Table 1:** Patients demographic and clinical characteristics.

Characteristics	Group A (PA-MSHA)	Group B (conservative)	*p*-value
Age (years), median (i.q.r)	35 (19–42)	38 (24–45)	0.109
Sex (Male/Female)	10/18	17/33	1.00
Length of stay (LOS)	7.57 ± 1.49	8.86 ± 0.38	0.034
Total drainage volume POD2	309.93 ± 45.21	243.78 ± 15.09	0.396
Total drainage volume POD3	91.50 ± 8.76	100.56 ± 4.42	0.309
POD3/POD2 rate	37%	44%	0.047
Total drainage volume*	401.43 ± 48.08	338.1 ± 17.64	0.414
Left drainage volume POD2	179.21 ± 34.16	113.86 ± 7.75	0.069
Left drainage volume POD3	46.89 ± 5.36	48.96 ± 3.27	0.624
Right drainage volume POD2	132.32 ± 16.67	129.92 ± 10.82	0.973
Right drainage volume POD3	44.61 ± 5.51	51.60 ± 3.76	0.243
Total drainage volume* (Left)	226.11 ± 36.12	162.82 ± 10.03	0.325
Total drainage volume* (Right)	176.93 ± 18.41	181.52 ± 14.10	0.973
Number of drug injections (=1/>1)	28/0	25/25 (50%)	0.001
Number of readmissions to hospital	2/28	15/50	0.022
Readmission rate	7.14%	30%	0.022
Fever	26/28	2/50	0.001
Neck pain	25/28	4/50	0.001

* Total drainage volume POD2 and POD3 combined. Before drug injection.

**Figure 5:**
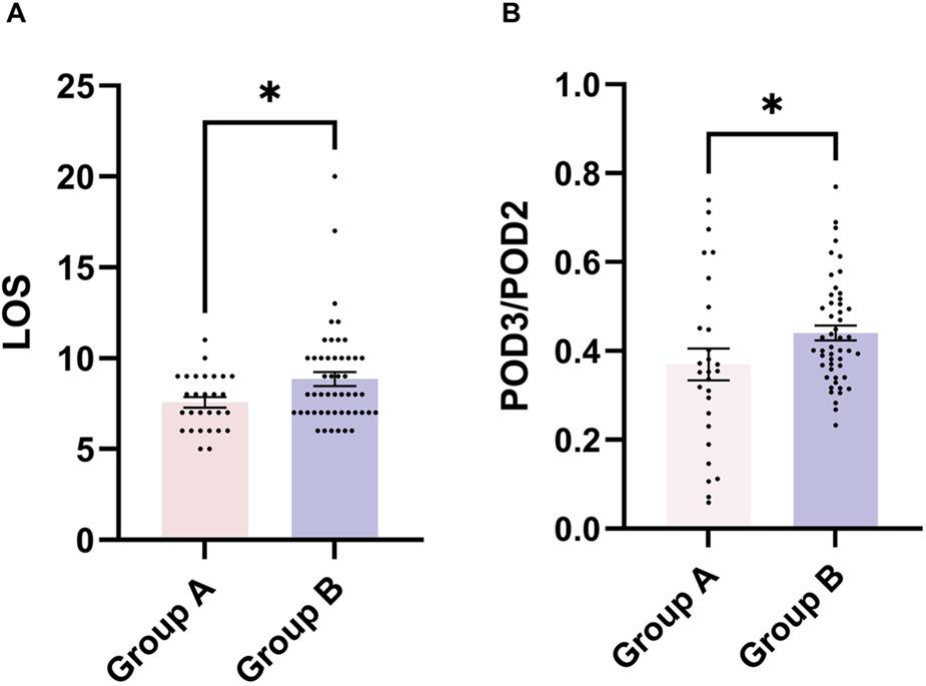
Outcomes of clinical data assessment of LOS **(A)** and POD3/POD2 ratios **(B)**. An asterisk indicates statistical significance (*p* < *0.05*).

In our clinical observation, all patients were successfully treated without requiring surgical re-intervention. Group A showed a visible reduction in chyle output after PA-MSHA injection, whereas patients in Group B often required repeated adhesion-agent injections and prolonged observation leading to a longer length of stay (7.57 ± 1.49 vs. 8.86 ± 0.38, *p* *=* *0.034*). No significant differences of age, sex, side of chyle leakage and drainage volume. Mean total drainage volume on either day in Group A was higher than that in Group B, while POD3/POD2 rate in Group A was lower than that in Group B (37% vs. 44%, *p* *=* *0.047*) (Figure 5). Readmission rate was 7.14% (Group A) vs. 30% (Group B).

### Propensity score and IPTW section

**Figure 6:**
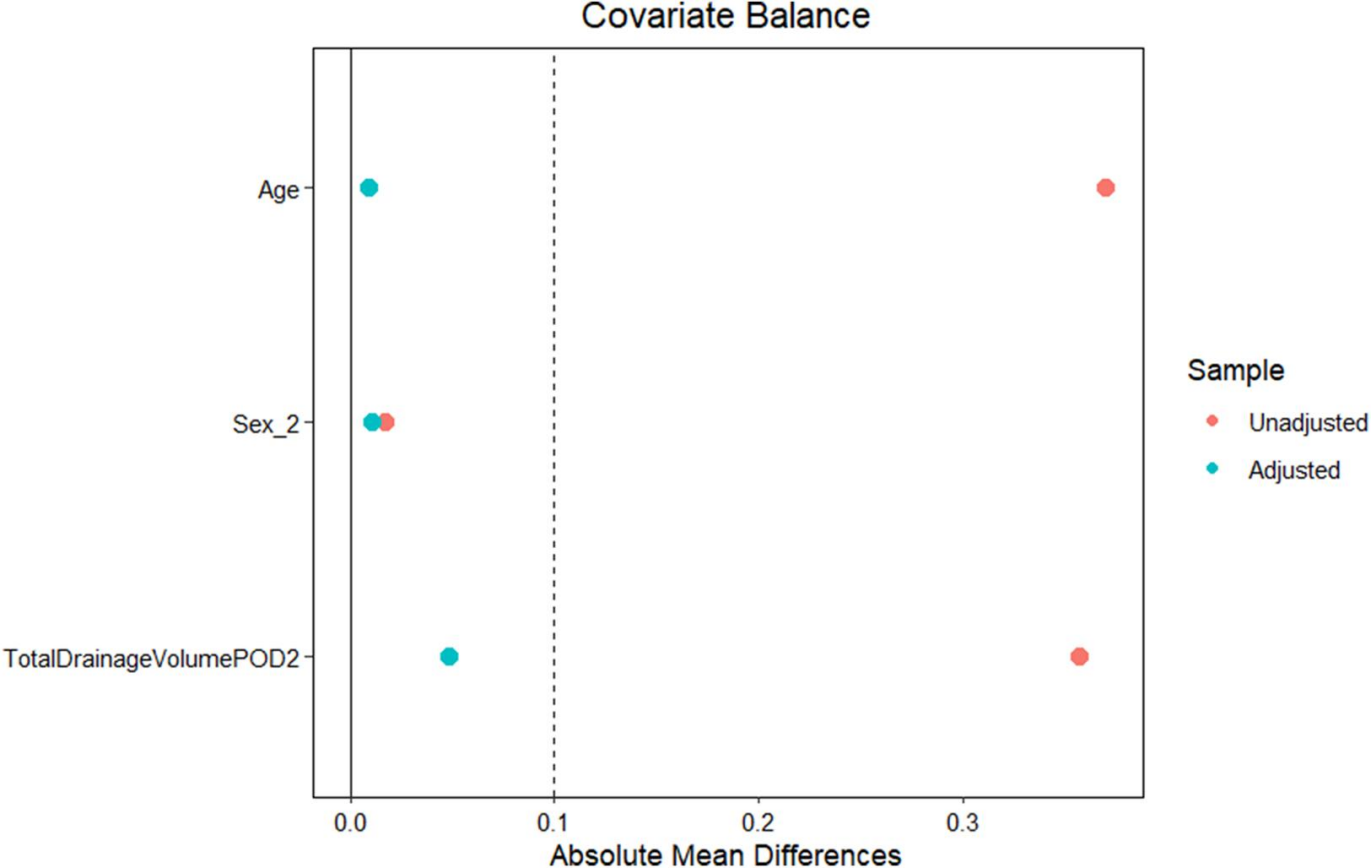
Covariate balance before and after inverse probability of treatment weighting (IPTW). Love plot displays absolute SMDs for baseline covariates. Red points represent unadjusted SMDs, and blue points represent SMDs after IPTW adjustment. The vertical dashed line indicates the conventional threshold of 0.1. All adjusted SMDs approached zero.

**Figure 7:**
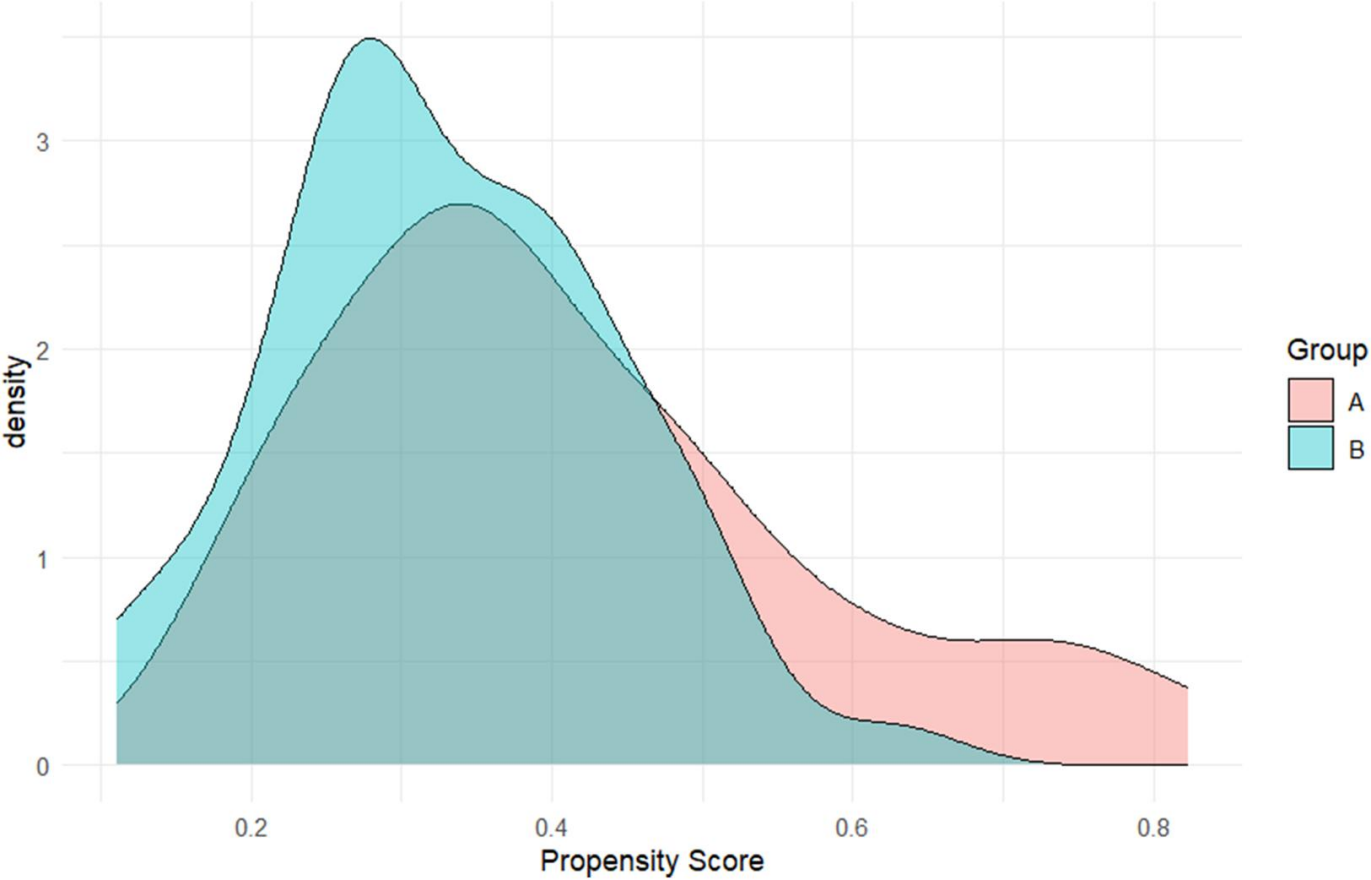
Distribution of propensity scores in two groups. Kernel density curves illustrate the distribution of estimated propensity scores. The two curves show adequate overlap, indicating sufficient common support and confirming that the propensity score model appropriately captured baseline differences between groups.

**Figure 8:**
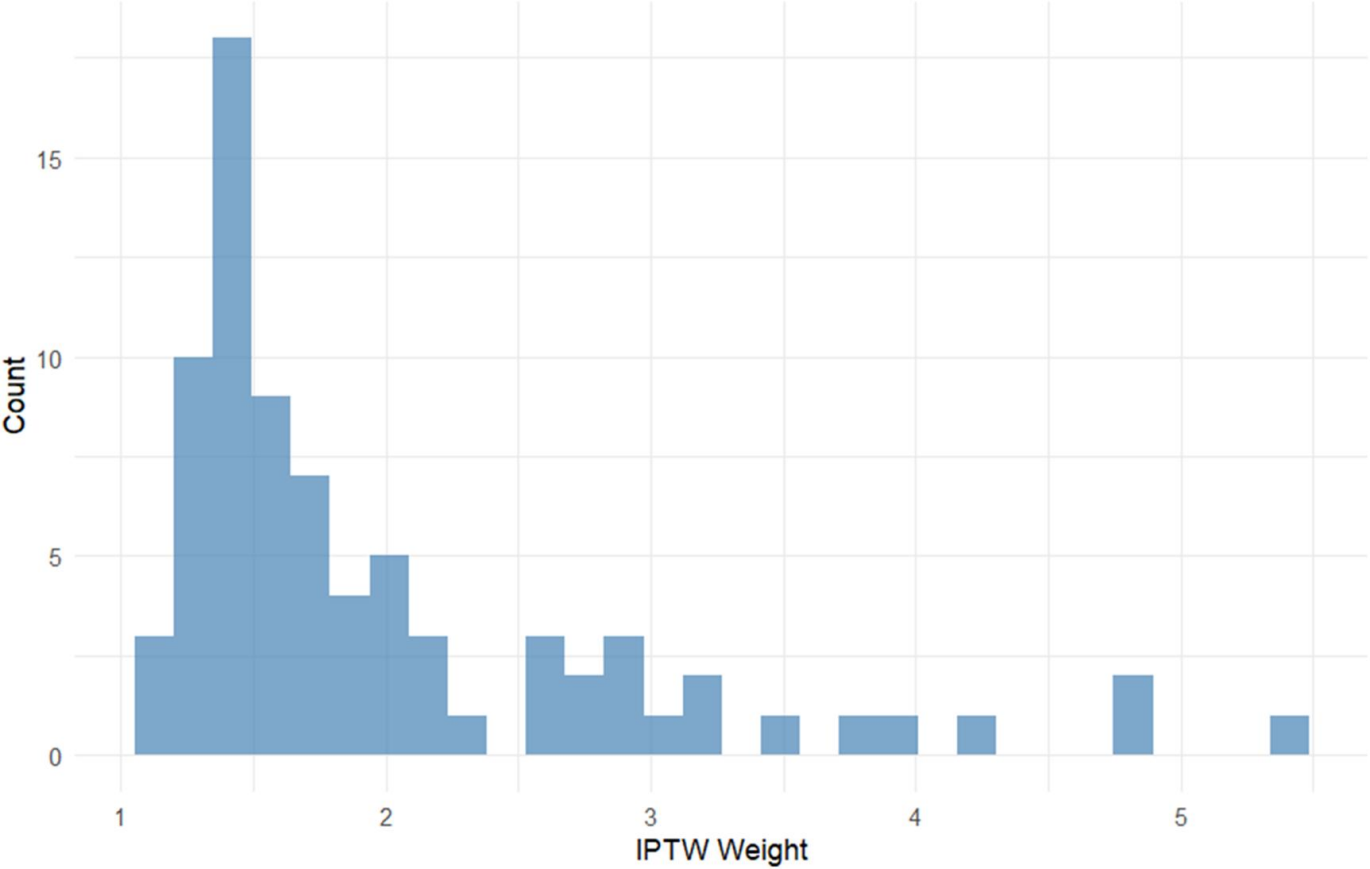
Distribution of inverse probability of treatment weights. The histogram shows the distribution of IPTW weights derived from the propensity score model.

After applying inverse probability of treatment weighting, baseline characteristics between Group A (PA-MSHA) and Group B (conservative) were well balanced. The weighted standardized mean differences for age, sex, and POD2 total drainage volume were all close to zero, which indicates a successful covariate balance (Figure 6). The distributions of propensity scores for the two groups demonstrated adequate overlap (Figure 7), and the IPTW weights showed a stable distribution without extreme values (Figure 8).

In weighted analysis, Group A demonstrated a significantly shorter LOS compared with Group B. Weighted linear regression showed that PA-MSHA treatment was associated with a mean reduction of 1.19 days in LOS (*β* = −1.19, *p* *=* *0.02*). No significant differences were observed in drainage-related secondary outcomes. POD3/POD2 rate was similar between groups (*β* = −0.033, *p* *=* *0.36*) and total drainage volume did not differ significantly (*β* = 3.30 mL, *p* *=* *0.93*).

Regarding postoperative adverse events, Group A showed higher rates of fever and neck pain. Weighted logistic regression indicated that PA-MSHA treatment was strongly associated with increased odds of fever (OR ≈ 266, *p* < 0.001) and neck pain (OR ≈ 66, *p* < 0.001). The model for the number of drug injections did not converge due to separation, and therefore no reliable IPTW-adjusted estimate could be obtained for this outcome.

### Major adverse events

Percutaneous PA-MSHA preparation wasn’t well tolerated by all patients. Major side effects associated with PA-MSHA injection were observed in most patients in Group A. Fever was reported by 26 patients (92.8%), and 25 patients (89.2%) suffered neck pain. A rapid increase in body temperature occurred in most patients within 2 to 4 h after the PA-MSHA injection, lasting 1 to 2 days. Neck pain was primarily reported around the drainage tube sites and percutaneous injection sites. Fever and neck pain were largely alleviated by oral loxoprofen sodium tablets (Daiichi-Sankyo Pharmaceutical Co. Ltd, Shanghai, China). All patients were periodically followed after discharge, with a mean follow-up period of 6 months (range: 1–12 months). No evidence of neck effusion, seroma, wound infection, dehiscence, or necrosis was found during the follow-up. No serious complications, such as drug allergies, sepsis, shock, or death, occurred.

## Discussion

Postoperative chyle fistula is an uncommon but clinically significant complication following total thyroidectomy and neck dissection. The incidence of chyle fistula varies with the extent of surgery, ranging from approximately 3% overall to 6%–8% in central and lateral dissection (15, 16). In thyroid surgery, chyle fistula most commonly occur in the left neck, particularly around the thoracic duct and its adjacent vessels (15). Therefore, meticulous intraoperative identification and ligation of the thoracic duct and its variant branches are crucial steps to prevent postoperative chyle fistula at the internal jugular angle (17, 18). Although advances in microsurgical techniques and energy devices have reduced the incidence of chyle leakage (16), small lymphatic channels may still reopen under elevated lymphatic pressure, leading to persistent low-output leakage (19, 20). This type of chyle fistula is characterized by prolonged drainage with a volume typically less than 500 mL per day (21).

Surgical intervention is recommended when drainage volume exceeds 1,000 mL per day, according to the literature, its outcomes are generally unsatisfactory (22). Conservative management remains the first-line treatment for postoperative chyle fistula, including dietary modification, local compression, negative-pressure drainage, and somatostatin administration (23). Local compression and negative-pressure drainage aimed to reduce the space around the lymphatic fistula. Diet modification and intravenous infusion of somatostatin (or its long-acting analogue) aimed to facilitate spontaneous fistula closure by reducing chyle production (24, 25). Conservative measures do not always achieve satisfactory outcomes, and prolonged leakage may delay recovery (18, 26). Therapeutic lymphography and thoracic duct embolization (TDE) have been considered as treatment modalities for chyle leak; however, these methods are not simple and not suitable for widespread promotion, particularly in small medical centers (27, 28).

In our study, patients in Group B received real-world clinical routine practice, reflecting a limited efficacy by longer hospitalization and higher POD3/POD2 drainage ratios. PA-MSHA has been reported to induce a localized inflammatory and fibrotic response that promotes sealing of lymphatic leaks (29, 30). Our clinical observations demonstrated a favorable therapeutic effect of PA-MSHA injection in both hospitalization and leakage decrease. Furthermore, in order to address baseline imbalances and a potential confounding of somatostatin effect in the control group, we applied inverse probability of treatment weighting (IPTW). After weighting, covariate balance between the two groups was substantially improved, as shown by the Love plot, propensity score overlap, and stable weight distribution. These findings help support the robustness of the adjusted comparisons.

In the IPTW-adjusted analysis, PA-MSHA treatment was associated with a significantly shorter hospitalization, suggesting that the intervention may accelerate the resolution of chyle leakage. Notably, our daily clinical observations showed that most patients in Group A experienced a visible reduction in chyle output within 24–48 h after PA-MSHA injection. This early trend did not translate into a statistically significant difference in the POD3/POD2 drainage reduction rate after IPTW adjustment, suggesting that the unadjusted association was largely driven by baseline imbalances, particularly the higher POD2 drainage volume in Group A, rather than by the treatment effect itself. Fever and neck pain were more common in the PA-MSHA group, likely reflecting the expected inflammatory response induced by inactivated bacterial components (31). These reactions were transient and manageable, and no severe complications were observed.

Nevertheless, our cohort study suggest that PA-MSHA may serve as a practical and accessible adjunct to conservative therapy for postoperative chyle fistula. In clinical settings where thoracic duct embolization or therapeutic lymphangiography is not readily available, PA-MSHA provides a minimally invasive alternative that can be performed under ultrasound guidance without specialized equipment (32). Although PA-MSHA did not significantly alter early postoperative drainage dynamics after IPTW adjustment, its association with a shorter hospitalization indicates that it may facilitate earlier clinical recovery and reduce the overall burden of care (33). These characteristics make PA-MSHA a potentially valuable option for managing low-output chyle fistula following lateral neck dissection.

Our study had several limitations. First, it was a retrospective single-center analysis with a modest sample size. Second, group allocation was based on patient preference and clinical judgment rather than randomization, introducing potential selection bias despite IPTW adjustment. Third, conservative treatment protocols in PA-MSHA experimental group may have potentially influenced outcomes, and future studies should optimize protocols to minimize confounding. Additionally, the optimal timing, dosage, and injection technique for PA-MSHA remain uncertain and warrant further investigation. Prospective studies, ideally randomized controlled trials, and standardized assessment tools are strongly needed to validate the therapeutic role of PA-MSHA, as well as to determine whether specific patient subgroups may benefit most from this intervention.

## Conclusion

Percutaneous ultrasound-guided PA-MSHA injection may serve as a practical adjunct to conservative therapy for postoperative chyle fistula following neck dissection. Associated with a significantly shorter hospitalization, PA-MSHA injection suggests a potential benefit in accelerating clinical recovery from thyroid surgery. Further prospective studies are needed to validate its efficacy and define its optimal clinical application.

## Data Availability

The original contributions presented in the study are included in the article/Supplementary Material, further inquiries can be directed to the corresponding author/s.
